# Crystal structure and Hirshfeld surface analysis of (2*E*)-1-(4-bromo­phen­yl)-3-(2-methyl­phen­yl)prop-2-en-1-one

**DOI:** 10.1107/S2056989023007387

**Published:** 2023-08-30

**Authors:** Mehmet Akkurt, Farid N. Naghiyev, Victor N. Khrustalev, Khammed A. Asadov, Ali N. Khalilov, Ajaya Bhattarai, İbrahim G. Mamedov

**Affiliations:** a Department of Physics, Faculty of Sciences, Erciyes University, 38039 Kayseri, Türkiye; b Department of Chemistry, Baku State University, Z. Khalilov str. 23, AZ1148, Baku, Azerbaijan; c Peoples’ Friendship University of Russia (RUDN University), Miklukho-Maklay St. 6, Moscow 117198, Russian Federation; d N. D. Zelinsky Institute of Organic Chemistry RAS, Leninsky Prosp. 47, Moscow, 119991, Russian Federation; e ‘Composite Materials’ Scientific Research Center, Azerbaijan State Economic University (UNEC), H. Aliyev str. 135, AZ1063, Baku, Azerbaijan; f Department of Chemistry, M.M.A.M.C. (Tribhuvan University) Biratnagar, Nepal; University of Neuchâtel, Switzerland

**Keywords:** crystal structure, *E* configuration, weak C—H⋯O inter­actions, face-to-face π–π stacking inter­actions, Hirshfeld surface analysis

## Abstract

In the title com­pound, C_16_H_13_BrO, the planes of the aromatic rings are inclined at an angle of 23.49 (15)°, and the configuration about the C=C bond is *E*. In the crystal, the mol­ecules are linked into chains by weak C—H⋯O inter­actions along the *b* axis. Successive chains form a zigzag structure along the *c* axis, and these chains are connected to each other by *face-to-face* π–π stacking inter­actions along the *a* axis. These layers, parallel to the (001) plane, are linked by van der Waals inter­actions, thus consolidating the crystal structure. Hirshfeld surface analysis showed that the most significant contacts in the structure are H⋯H (43.1%), C⋯H/H⋯C (17.4%), Br⋯H/H⋯Br (14.9%), C⋯C (11.9%) and O⋯H/H⋯O (9.8%).

## Abstract 2

In the crystal of (2*E*)-1-(4-bromo­phen­yl)-3-(2-methyl­phen­yl)prop-2-en-1-one, the mol­ecules are linked into chains by weak C—H⋯O inter­actions along the *b* axis. Successive chains form a zigzag structure along the *c* axis, and these chains are connected to each other by *face-to-face* π–π stacking inter­actions along the *a* axis, forming layers parallel to the (001) plane. The crystal structure maintains its stability *via* van der Waals inter­actions between the layers.

## 1. Chemical context

Diverse C—C, C—N, C—S and C—O bond formations are fundamental and valuable conversions in modern organic chemistry (Gurbanov *et al.*, 2017 ▸; Afkhami *et al.*, 2019 ▸; Mahmoudi *et al.*, 2021 ▸). Chalcones are α,β-unsaturated ketones containing ar­yl–aryl or ar­yl–alkyl groups at both ends. They belong to the flavonoid family, and they possess a wide variety of biological activities. Many natural chalcones, such as echinatin, naringenin, isoliquiritigenin, butein, 4-hy­droxy­derricin, 4-hy­droxy­lonchocarpin, derricin, xanthoangelol, lonchocarpin, licochalcone A, licochalcone E, humulusol, munsericin, flavokawain A, isobavachalcone, mallotophilippen C, D and E, broussochalcone A, crotaorixin, pedicinin and nardoaristolone A have been isolated from plants (Rozmer & Perjési, 2016 ▸; Çelik *et al.*, 2023 ▸; Chalkha *et al.*, 2023 ▸). Moreover, the enone moiety is a widespread structural motif often found in biologically active com­pounds possessing enzyme inhibitory, anti­cancer and anti­microbial activity (Poustforoosh *et al.*, 2022 ▸; Tapera *et al.*, 2022 ▸; Sarkı *et al.*, 2023 ▸). Herein, in continuation to our recent investigations (Gurbanov *et al.*, 2022*a* ▸,*b* ▸), we report the crystal structure and Hirshfeld surface analysis of (2*E*)-1-(4-bromo­phen­yl)-3-(2-methyl­phen­yl)prop-2-en-1-one. 
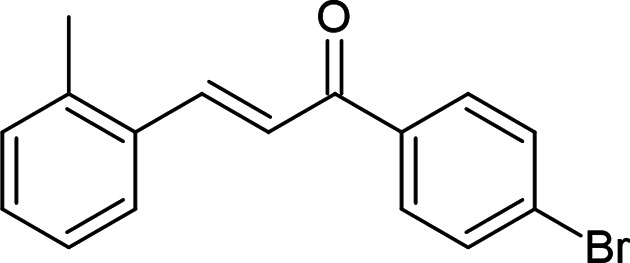


## 2. Structural commentary

**Figure 1:**
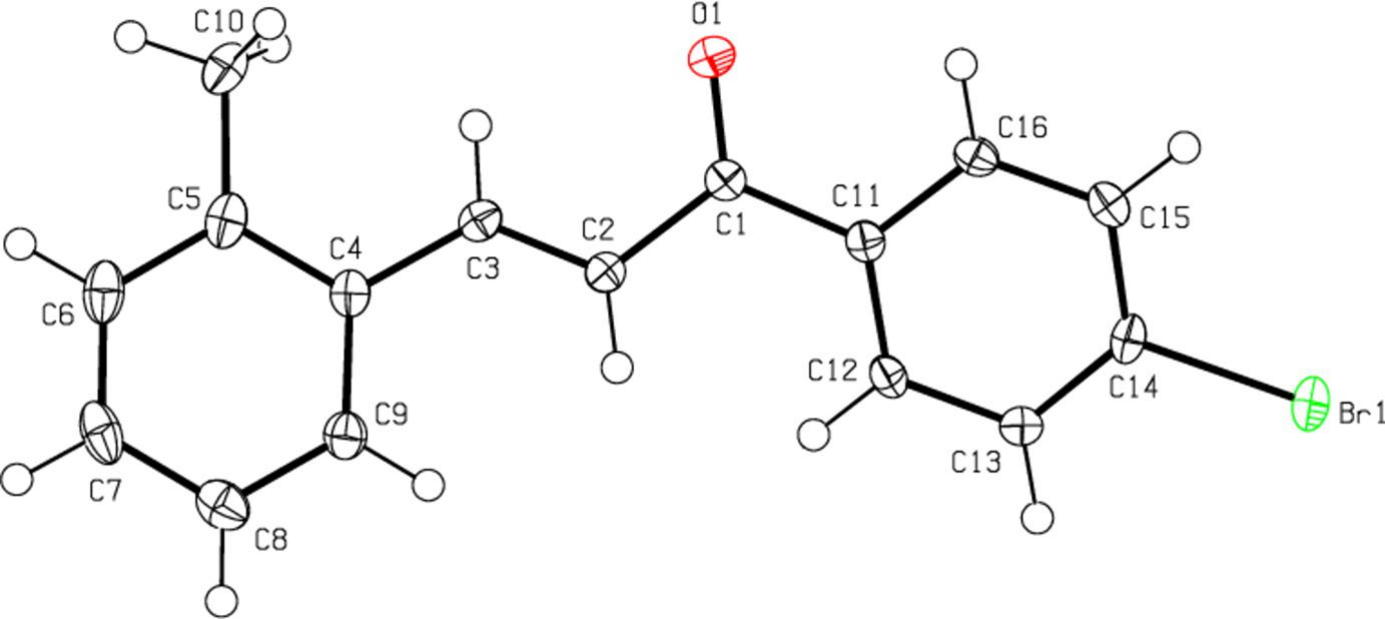
The mol­ecular structure of the title com­pound, showing the atom labelling and displacement ellipsoids drawn at the 50% probability level.

The title com­pound (Fig. 1 ▸) is com­posed of two aromatic rings, *i.e.* 2-methyl­phenyl (C4–C9) and 4-bromo­phenyl (C11–C16), which are linked by a –CO—CH=CH– *E*-configured enone bridge. The mol­ecule is approximately planar, as indicated by the torsion angles C10—C5—C4—C3 = 1.9 (5)°, C9—C4—C3—C2 = −4.4 (5)°, C4—C3—C2—C1 = −176.3 (3)°, C3—C2—C1—C11 = −168.2 (3)°, C2—C1—C11—C12 = 15.9 (4)° and Br1—C14—C15—C16 = 178.5 (2)°. The dihedral angle between the planes of the 2-methyl­phenyl and 4-bromo­phenyl rings is 23.49 (15)°.

## 3. Supra­molecular features and Hirshfeld surface analysis

**Figure 2:**
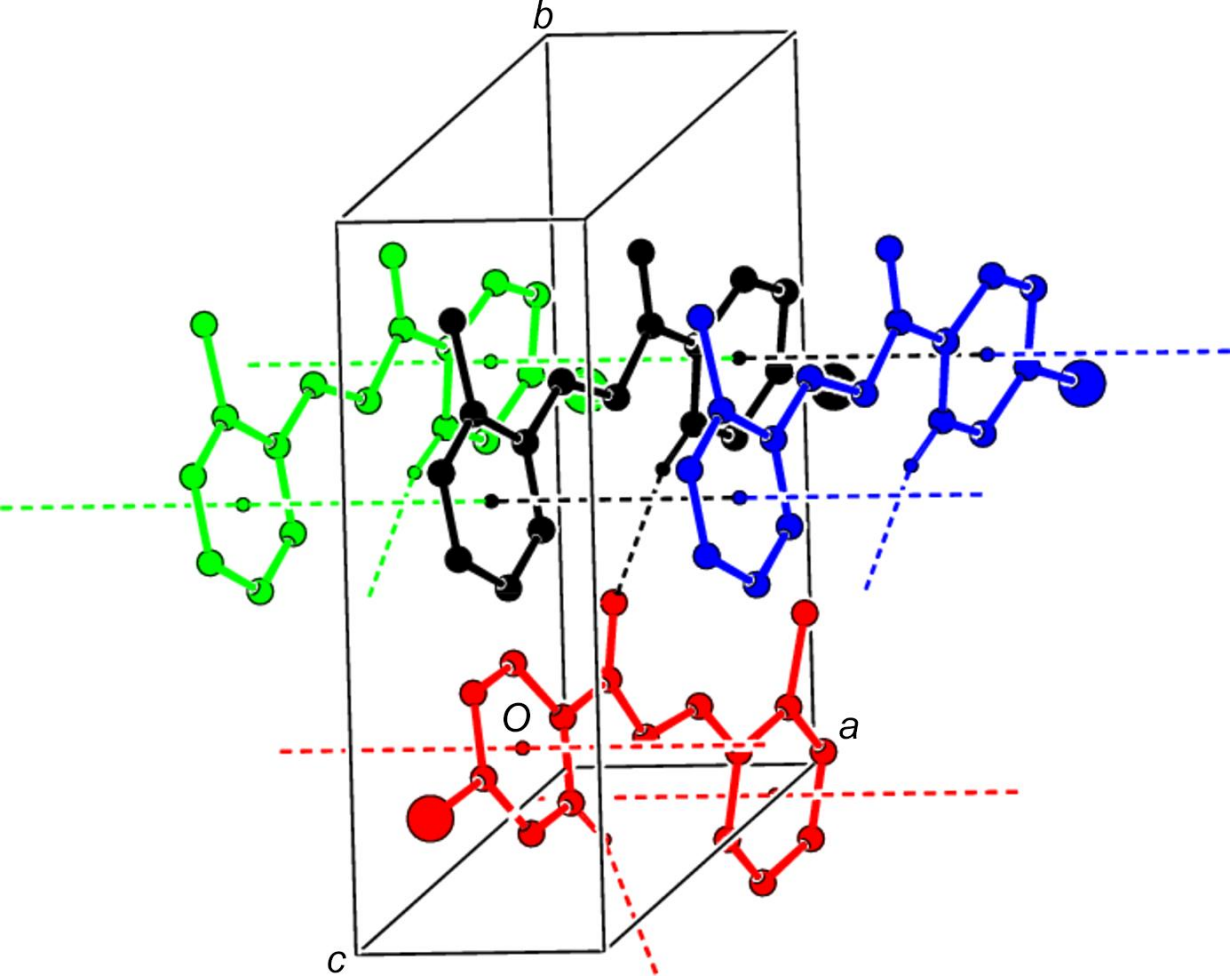
View of the C—H⋯O hydrogen bonds and *face-to-face* π–π stacking inter­actions in the title com­pound along the *c* axis.

**Figure 3:**
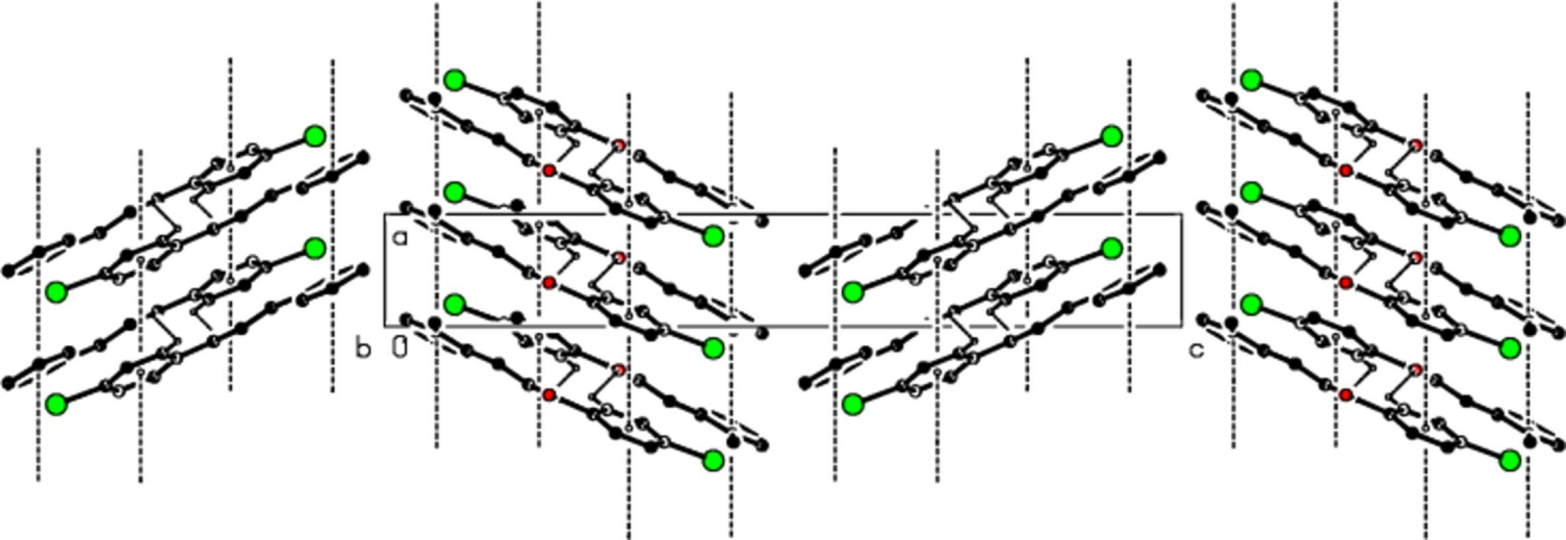
Zigzag packing of the title com­pound along the *b* axis.

In the crystal, the mol­ecules are linked into *C*(5) chains (Bernstein *et al.*, 1995 ▸) by weak C—H⋯O inter­actions (Table 1 ▸ and Fig. 2 ▸) along the *a* axis. Successive chains form a zigzag structure along the *b* axis (Fig. 3 ▸) and these chains are connected to each other along the *c* axis by *face-to-face* π–π stacking inter­actions [*Cg*1⋯*Cg*1^*a*^ = 3.942 (2) Å, slippage = 1.890 Å; *Cg*2⋯*Cg*2^*a*^ = 3.9420 (18) Å, slippage = 1.942 Å; symmetry code: (*a*) *x* − 1, *y*, *z*; *Cg*1 and *Cg*2 are the centroids of the 2-methyl­phenyl (C4—C9) and 4-bromo­phenyl (C11–C16) rings, respectively]. They form layers parallel to the (001) plane through van der Waals inter­actions, thus consolidating the crystal structure.

*CrystalExplorer17.5* (Spackman *et al.*, 2021 ▸) was used to com­pute the Hirshfeld surfaces and the two-dimensional fingerprints of the title mol­ecule. The *d*_norm_ mappings for the title com­pound were performed in the range from −0.0627 to +1.1373 a.u., on the *d*_norm_ surfaces, allowing the location of the C—H⋯O inter­actions (Tables 1 ▸ and 2 ▸).

**Figure 4:**
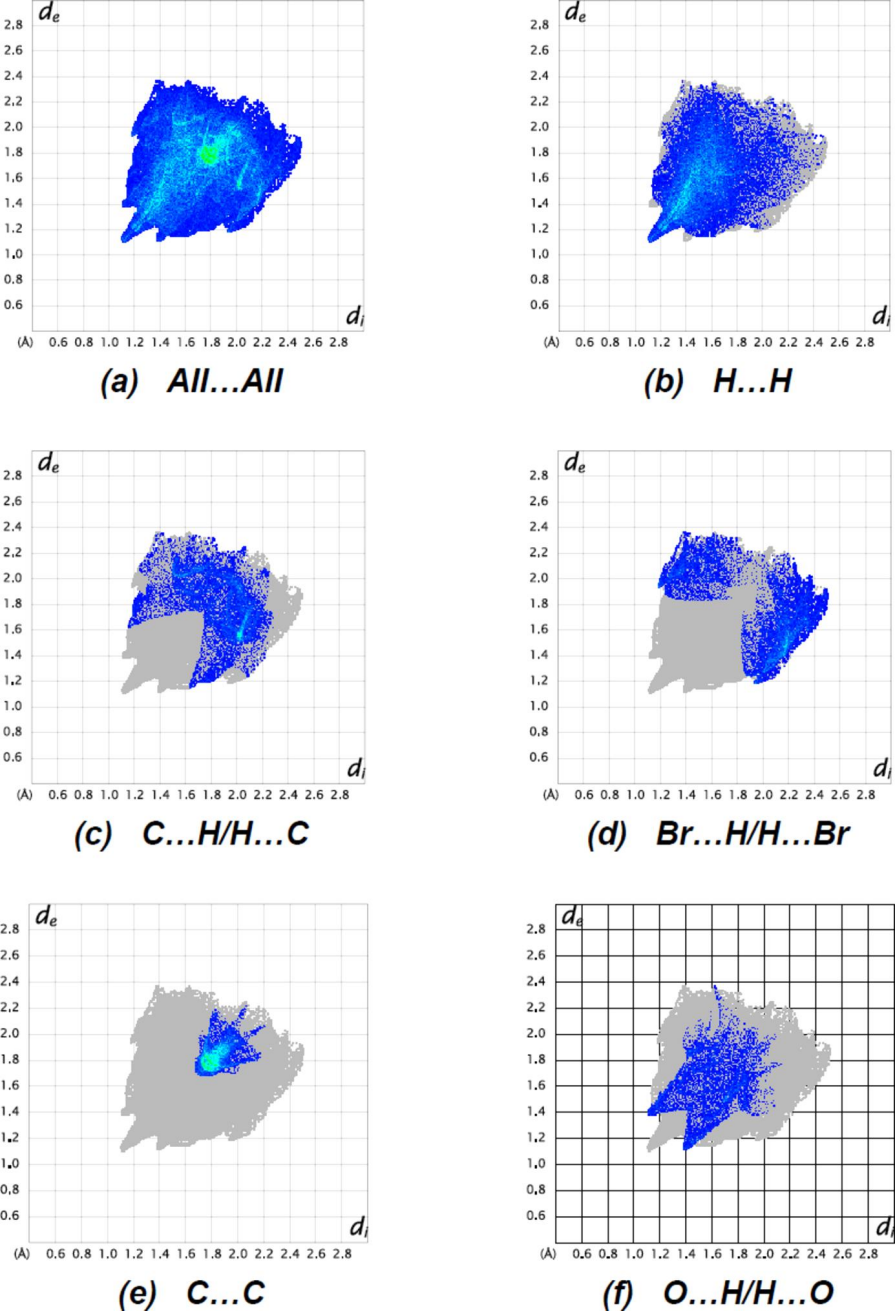
The two-dimensional fingerprint plots of the title com­pound, showing (*a*) all inter­actions, and delineated into (*b*) H⋯H, (*c*) C⋯H/H⋯C, (*d*) Br⋯H/H⋯Br, (*e*) C⋯C and (*f*) O⋯H/H⋯O inter­actions. *d*_e_ and *d*_i_ represent the distances from a point on the Hirshfeld surface to the nearest atoms outside (external) and inside (inter­nal) the surface.

The fingerprint plots (Fig. 4 ▸) show that H⋯H [Fig. 4 ▸(*b*); 43.1%], C⋯H/H⋯C [Fig. 4 ▸(*c*); 17.4%], Br⋯H/H⋯Br [Fig. 4 ▸(*d*); 14.9%], C⋯C [Fig. 4 ▸(*e*); 11.9%] and O⋯H/H⋯O [Fig. 4 ▸(*f*); 9.8%] inter­actions contribute the most to the surface contacts. The crystal packing is additionally influenced by Br⋯C/C⋯Br (2.0%), Br⋯Br (0.8%), N⋯N (2.6%) and O⋯C/C⋯O (0.2%) contacts. The Hirshfeld surface study confirms the significance of H-atom inter­actions in the packing formation. The large number of H⋯H, C⋯H/H⋯C, Br⋯H/H⋯Br, C⋯C and O⋯H/H⋯O inter­actions indicates that van der Waals inter­actions and hydrogen bonding are important in the crystal packing (Hathwar *et al.*, 2015 ▸).

## 4. Database survey

Four related com­pounds were found as a result of a search for the ‘(2*E*)-1,3-di­phenyl­prop-2-en-1-one’ unit in the Cambridge Structural Database (CSD, Version 5.42, update of September 2021; Groom *et al.*, 2016 ▸), *viz.* CSD refcodes KOCZUA (Bindya *et al.*, 2019 ▸), RUCKIM (Spruce *et al.*, 2020 ▸), XOLLOC (Çelikesir *et al.*, 2019 ▸) and OBIYUW01 (Atioğlu *et al.*, 2019 ▸).

In the crystal of KOCZUA, the shortest inter­molecular contacts are Cl⋯O [3.173 (3) Å]; these link the mol­ecules to form a 2_1_ helix propagating along the *b*-axis direction. The helices are linked by offset π–π inter­actions [inter­centroid distance = 3.983 (1) Å], forming layers lying parallel to the *ab* plane. In the crystal of RUCKIM, the mol­ecules are linked through type II halogen bonds, forming a sheet structure parallel to the *bc* plane. Weak inter­molecular C—H⋯π inter­actions are observed between the sheets. In the crystal of XOLLOC, mol­ecules are linked *via* pairs of C—H⋯O inter­actions with an 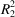 (14) ring motif, forming inversion dimers. The dimers are linked into a tape structure running along [101] *via* C—H⋯π inter­actions. In the crystal of OBIYUW01, mol­ecules are linked by C—H⋯π inter­actions between the bromo­phenyl and fluoro­phenyl rings, resulting in a two-dimensional layered structure parallel to the *ab* plane. The mol­ecular packing is consolidated by weak Br⋯H and F⋯H contacts.

## 5. Synthesis and crystallization

The title com­pound was synthesized using a reported procedure (Chithiraikumar *et al.*, 2021 ▸) and colourless crystals were obtained upon recrystallization from an ethanol/water (3:1 *v*/*v*) solution at room temperature.

## 6. Refinement

Crystal data, data collection and structure refinement details are summarized in Table 3 ▸. All H atoms were placed in their geometrically calculated positions and refined using a riding model, with C—H = 0.95 Å and *U*_iso_(H) = 1.2*U*_eq_(C) for aromatic H atoms, and C—H = 0.98 Å and *U*_iso_(H) = 1.5*U*_eq_(C) for methyl H atoms.

## Supplementary Material

Crystal structure: contains datablock(s) I, global. DOI: 10.1107/S2056989023007387/tx2073sup1.cif

Structure factors: contains datablock(s) I. DOI: 10.1107/S2056989023007387/tx2073Isup2.hkl

CCDC reference: 2290092

Additional supporting information:  crystallographic information; 3D view; checkCIF report

## Figures and Tables

**Table 1:** Hydrogen-bond geometry (Å, °)

*D*—H⋯*A*	*D*—H	H⋯*A*	*D*⋯*A*	*D*—H⋯*A*
C12—H12⋯O1^i^	0.95	2.58	3.200 (3)	124

Symmetry code: (i) 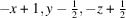 .

**Table 2:** Summary of short inter­atomic contacts (Å) in the title com­pound

C3⋯H10*B*	2.85	*x* + 1, *y*, *z*
Br1⋯H7	3.17	−*x* + 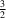 , −*y* + 1, *z* − 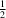
O1⋯H12	2.58	−*x* + 1, *y* + 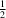 , −*z* + 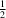
H15⋯H9	2.45	−*x* + 2, *y* + 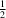 , −*z* + 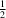
C10⋯H10*A*	3.10	*x* + 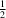 , −*y* + 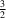 , −*z* + 1

**Table 3:** Experimental details

Crystal data	
Chemical formula	C_16_H_13_BrO
*M*_r_	301.17
Crystal system, space group	Orthorhombic, *P*2_1_2_1_2_1_
Temperature (K)	100
*a*, *b*, *c* (Å)	3.942, 11.5915 (2), 28.0387 (4)
*V* (Å^3^)	1281.19 (3)
*Z*	4
Radiation type	Cu *K*α
μ (mm^−1^)	4.23
Crystal size (mm)	0.35 × 0.09 × 0.07

Data collection	
Diffractometer	Rigaku XtaLAB Synergy Dualflex diffractometer with a HyPix detector
Absorption correction	Gaussian (*CrysAlis PRO*; Rigaku OD, 2022 ▸)
*T*_min_, *T*_max_	0.251, 1.000
No. of measured, independent and observed [*I* > 2σ(*I*)] reflections	14537, 2657, 2627
*R*_int_	0.025
(sin θ/λ)_max_ (Å^−1^)	0.632

Refinement	
*R*[*F* ^2^ > 2σ(*F* ^2^)], *wR*(*F* ^2^), *S*	0.022, 0.052, 1.14
No. of reflections	2657
No. of parameters	165
H-atom treatment	H-atom parameters constrained
Δρ_max_, Δρ_min_ (e Å^−3^)	0.51, −0.42
Absolute structure	Refined as an inversion twin
Absolute structure parameter	0.53 (2)

Computer programs: *CrysAlis PRO* (Rigaku OD, 2022 ▸), *SHELXT2018* (Sheldrick, 2015*a* ▸), *SHELXL2018* (Sheldrick, 2015*b* ▸), *ORTEP-3 for Windows* (Farrugia, 2012 ▸) and *PLATON* (Spek, 2020 ▸).

## supplementary crystallographic information

### Crystal data

**Table:**

C_16_H_13_BrO	*D*_x_ = 1.561 Mg m^−^^3^
*M**_r_* = 301.17	Cu *K*α radiation, λ = 1.54184 Å
Orthorhombic, *P*2_1_2_1_2_1_	Cell parameters from 12560 reflections
*a* = 3.942 Å	θ = 3.8–76.9°
*b* = 11.5915 (2) Å	µ = 4.23 mm^−^^1^
*c* = 28.0387 (4) Å	*T* = 100 K
*V* = 1281.19 (3) Å^3^	Needle, colourless
*Z* = 4	0.35 × 0.09 × 0.07 mm
*F*(000) = 608	

### Data collection

**Table:**

Rigaku XtaLAB Synergy Dualflex diffractometer with a HyPix detector	2627 reflections with *I* > 2σ(*I*)
Radiation source: micro-focus sealed X-ray tube	*R*_int_ = 0.025
φ and ω scans	θ_max_ = 77.1°, θ_min_ = 3.2°
Absorption correction: gaussian (CrysAlis PRO; Rigaku OD, 2022)	*h* = −4→4
*T*_min_ = 0.251, *T*_max_ = 1.000	*k* = −14→14
14537 measured reflections	*l* = −35→34
2657 independent reflections	

### Refinement

**Table:**

Refinement on *F*^2^	Hydrogen site location: inferred from neighbouring sites
Least-squares matrix: full	H-atom parameters constrained
*R*[*F*^2^ > 2σ(*F*^2^)] = 0.022	*w* = 1/[σ^2^(*F*_o_^2^) + (0.0121*P*)^2^ + 1.3924*P*] where *P* = (*F*_o_^2^ + 2*F*_c_^2^)/3
*wR*(*F*^2^) = 0.052	(Δ/σ)_max_ = 0.001
*S* = 1.14	Δρ_max_ = 0.51 e Å^−^^3^
2657 reflections	Δρ_min_ = −0.42 e Å^−^^3^
165 parameters	Absolute structure: Refined as an inversion twin
0 restraints	Absolute structure parameter: 0.53 (2)

### Special details

**Table:**

Geometry. All esds (except the esd in the dihedral angle between two l.s. planes) are estimated using the full covariance matrix. The cell esds are taken into account individually in the estimation of esds in distances, angles and torsion angles; correlations between esds in cell parameters are only used when they are defined by crystal symmetry. An approximate (isotropic) treatment of cell esds is used for estimating esds involving l.s. planes.
Refinement. Refined as a 2-component inversion twin.

### Fractional atomic coordinates and isotropic or equivalent isotropic displacement parameters (Å^2^)

**Table:**

	*x*	*y*	*z*	*U*_iso_*/*U*_eq_	
C1	0.6338 (7)	0.6732 (2)	0.28488 (10)	0.0156 (6)	
C2	0.5186 (8)	0.5849 (3)	0.31975 (11)	0.0179 (6)	
H2	0.576288	0.506108	0.315259	0.021*	
C3	0.3333 (8)	0.6169 (3)	0.35751 (10)	0.0167 (6)	
H3	0.272264	0.696111	0.359037	0.020*	
C4	0.2138 (8)	0.5441 (3)	0.39673 (9)	0.0162 (5)	
C5	0.0419 (8)	0.5941 (3)	0.43586 (11)	0.0199 (6)	
C6	−0.0614 (9)	0.5231 (3)	0.47322 (11)	0.0243 (7)	
H6	−0.174643	0.556148	0.499821	0.029*	
C7	−0.0025 (9)	0.4050 (3)	0.47241 (12)	0.0260 (8)	
H7	−0.075592	0.358302	0.498276	0.031*	
C8	0.1629 (10)	0.3552 (3)	0.43386 (11)	0.0244 (7)	
H8	0.202810	0.274348	0.433117	0.029*	
C9	0.2691 (8)	0.4245 (3)	0.39655 (10)	0.0200 (6)	
H9	0.382068	0.390249	0.370170	0.024*	
C10	−0.0317 (9)	0.7216 (3)	0.43748 (12)	0.0225 (7)	
H10A	−0.155611	0.739898	0.466828	0.034*	
H10B	−0.169550	0.743225	0.409802	0.034*	
H10C	0.182210	0.764687	0.436880	0.034*	
C11	0.7811 (8)	0.6341 (2)	0.23829 (10)	0.0151 (5)	
C12	0.7419 (7)	0.5219 (2)	0.22102 (9)	0.0170 (6)	
H12	0.629276	0.465770	0.239962	0.020*	
C13	0.8664 (8)	0.4916 (2)	0.17629 (11)	0.0180 (6)	
H13	0.839832	0.415359	0.164401	0.022*	
C14	1.0295 (8)	0.5750 (3)	0.14959 (10)	0.0170 (6)	
C15	1.0768 (8)	0.6869 (3)	0.16597 (11)	0.0178 (6)	
H15	1.193503	0.742210	0.147111	0.021*	
C16	0.9500 (8)	0.7159 (3)	0.21041 (11)	0.0172 (6)	
H16	0.978056	0.792262	0.222085	0.021*	
Br1	1.19480 (8)	0.53645 (3)	0.08770 (2)	0.02188 (9)	
O1	0.6109 (6)	0.77583 (19)	0.29371 (8)	0.0242 (5)	

### Atomic displacement parameters (Å^2^)

**Table:**

	*U*^11^	*U*^22^	*U*^33^	*U*^12^	*U*^13^	*U*^23^
C1	0.0136 (15)	0.0168 (14)	0.0164 (13)	0.0005 (11)	−0.0014 (11)	0.0006 (11)
C2	0.0207 (16)	0.0164 (14)	0.0165 (14)	0.0013 (12)	0.0014 (12)	−0.0001 (11)
C3	0.0164 (13)	0.0177 (13)	0.0160 (13)	0.0015 (13)	−0.0028 (12)	−0.0018 (11)
C4	0.0129 (13)	0.0224 (13)	0.0134 (11)	−0.0004 (13)	−0.0030 (9)	0.0003 (11)
C5	0.0137 (14)	0.0316 (18)	0.0145 (14)	−0.0011 (13)	−0.0031 (12)	−0.0034 (13)
C6	0.0193 (14)	0.040 (2)	0.0142 (13)	−0.0042 (16)	−0.0013 (11)	−0.0015 (14)
C7	0.0249 (18)	0.038 (2)	0.0153 (15)	−0.0111 (15)	−0.0042 (13)	0.0066 (14)
C8	0.0264 (17)	0.0242 (16)	0.0226 (15)	−0.0024 (15)	−0.0079 (15)	0.0043 (12)
C9	0.0205 (17)	0.0229 (14)	0.0166 (13)	0.0004 (12)	−0.0016 (11)	0.0005 (10)
C10	0.0194 (16)	0.0280 (17)	0.0203 (16)	0.0003 (14)	0.0009 (13)	−0.0072 (13)
C11	0.0128 (13)	0.0171 (13)	0.0155 (13)	0.0022 (11)	−0.0008 (11)	0.0023 (10)
C12	0.0162 (15)	0.0178 (14)	0.0170 (12)	−0.0007 (12)	0.0021 (10)	0.0043 (11)
C13	0.0200 (16)	0.0151 (14)	0.0190 (14)	0.0005 (11)	0.0005 (12)	−0.0003 (10)
C14	0.0133 (14)	0.0253 (16)	0.0123 (13)	0.0024 (11)	0.0009 (11)	−0.0012 (11)
C15	0.0160 (14)	0.0194 (15)	0.0181 (14)	−0.0021 (12)	−0.0007 (11)	0.0042 (11)
C16	0.0172 (14)	0.0151 (14)	0.0194 (14)	−0.0023 (12)	−0.0019 (12)	0.0009 (11)
Br1	0.02033 (14)	0.03011 (16)	0.01522 (13)	−0.00038 (13)	0.00375 (12)	−0.00153 (13)
O1	0.0303 (14)	0.0171 (11)	0.0252 (11)	0.0010 (9)	0.0064 (10)	−0.0025 (9)

### Geometric parameters (Å, º)

**Table:**

C1—O1	1.218 (4)	C8—H8	0.9500
C1—C2	1.487 (4)	C9—H9	0.9500
C1—C11	1.500 (4)	C10—H10A	0.9800
C2—C3	1.339 (4)	C10—H10B	0.9800
C2—H2	0.9500	C10—H10C	0.9800
C3—C4	1.464 (4)	C11—C12	1.396 (4)
C3—H3	0.9500	C11—C16	1.398 (4)
C4—C9	1.404 (4)	C12—C13	1.392 (4)
C4—C5	1.414 (4)	C12—H12	0.9500
C5—C6	1.393 (5)	C13—C14	1.381 (4)
C5—C10	1.507 (5)	C13—H13	0.9500
C6—C7	1.388 (5)	C14—C15	1.389 (4)
C6—H6	0.9500	C14—Br1	1.907 (3)
C7—C8	1.389 (5)	C15—C16	1.384 (4)
C7—H7	0.9500	C15—H15	0.9500
C8—C9	1.384 (4)	C16—H16	0.9500
			
O1—C1—C2	121.1 (3)	C4—C9—H9	119.2
O1—C1—C11	120.1 (3)	C5—C10—H10A	109.5
C2—C1—C11	118.9 (3)	C5—C10—H10B	109.5
C3—C2—C1	119.7 (3)	H10A—C10—H10B	109.5
C3—C2—H2	120.1	C5—C10—H10C	109.5
C1—C2—H2	120.1	H10A—C10—H10C	109.5
C2—C3—C4	127.6 (3)	H10B—C10—H10C	109.5
C2—C3—H3	116.2	C12—C11—C16	119.4 (3)
C4—C3—H3	116.2	C12—C11—C1	122.8 (3)
C9—C4—C5	118.8 (3)	C16—C11—C1	117.8 (3)
C9—C4—C3	121.1 (3)	C13—C12—C11	120.6 (3)
C5—C4—C3	120.1 (3)	C13—C12—H12	119.7
C6—C5—C4	118.8 (3)	C11—C12—H12	119.7
C6—C5—C10	120.0 (3)	C14—C13—C12	118.4 (3)
C4—C5—C10	121.2 (3)	C14—C13—H13	120.8
C7—C6—C5	121.4 (3)	C12—C13—H13	120.8
C7—C6—H6	119.3	C13—C14—C15	122.5 (3)
C5—C6—H6	119.3	C13—C14—Br1	119.2 (2)
C6—C7—C8	120.1 (3)	C15—C14—Br1	118.3 (2)
C6—C7—H7	119.9	C16—C15—C14	118.4 (3)
C8—C7—H7	119.9	C16—C15—H15	120.8
C9—C8—C7	119.3 (3)	C14—C15—H15	120.8
C9—C8—H8	120.4	C15—C16—C11	120.7 (3)
C7—C8—H8	120.4	C15—C16—H16	119.7
C8—C9—C4	121.6 (3)	C11—C16—H16	119.7
C8—C9—H9	119.2		
			
O1—C1—C2—C3	12.0 (5)	C3—C4—C9—C8	178.7 (3)
C11—C1—C2—C3	−168.2 (3)	O1—C1—C11—C12	−164.3 (3)
C1—C2—C3—C4	−176.3 (3)	C2—C1—C11—C12	15.9 (4)
C2—C3—C4—C9	−4.4 (5)	O1—C1—C11—C16	12.6 (4)
C2—C3—C4—C5	175.1 (3)	C2—C1—C11—C16	−167.2 (3)
C9—C4—C5—C6	1.1 (5)	C16—C11—C12—C13	−0.6 (4)
C3—C4—C5—C6	−178.4 (3)	C1—C11—C12—C13	176.2 (3)
C9—C4—C5—C10	−178.6 (3)	C11—C12—C13—C14	0.1 (4)
C3—C4—C5—C10	1.9 (5)	C12—C13—C14—C15	0.8 (5)
C4—C5—C6—C7	−0.8 (5)	C12—C13—C14—Br1	−178.8 (2)
C10—C5—C6—C7	178.9 (3)	C13—C14—C15—C16	−1.1 (5)
C5—C6—C7—C8	0.1 (6)	Br1—C14—C15—C16	178.5 (2)
C6—C7—C8—C9	0.3 (5)	C14—C15—C16—C11	0.6 (5)
C7—C8—C9—C4	0.1 (5)	C12—C11—C16—C15	0.3 (5)
C5—C4—C9—C8	−0.7 (5)	C1—C11—C16—C15	−176.7 (3)

### Hydrogen-bond geometry (Å, º)

**Table:**

*D*—H···*A*	*D*—H	H···*A*	*D*···*A*	*D*—H···*A*
C3—H3···O1	0.95	2.45	2.791 (4)	101
C12—H12···O1^i^	0.95	2.58	3.200 (3)	124

Symmetry code: (i) −*x*+1, *y*−1/2, −*z*+1/2.
